# Co‐construction of health technology assessment recommendations with patients: An example with cardiac defibrillator replacement

**DOI:** 10.1111/hex.12989

**Published:** 2019-11-05

**Authors:** Marie‐Pascale Pomey, Philippe Brouillard, Isabelle Ganache, Laurie Lambert, Lucy Boothroyd, Caroline Collette, Sylvain Bédard, Alexandre Grégoire, Sandra Pelaez, Olivier Demers‐Payette, Mireille Goetghebeur, Michèle de Guise, Denis Roy

**Affiliations:** ^1^ School of Public Health Université de Montréal Montreal Québec Canada; ^2^ Institut national d’excellence en santé et services sociaux Montreal Québec Canada; ^3^ Center of Excellence on Partnership with Patients and the Public Montreal Québec Canada; ^4^ Centre hospitalier universitaire l’université de Montréal research center (CRCHUM) Montreal Québec Canada; ^5^ Faculty of Medicine Montreal Québec Canada; ^6^ McGill University Montreal Québec Canada

**Keywords:** co‐construction, HTA, ICD, INESSS, patient engagement, patient involvement, recommendations

## Abstract

**Context:**

The National Institute of Excellence in Health and Social Services (INESSS), which functions as the Québec health technology assessment (HTA) agency, tested a new way to engage patients along with health‐care professionals in the co‐construction of recommendations regarding implantable cardioverter‐defibrillator replacement.

**Objective:**

The objective of this article was to describe the process of co‐construction of recommendations and to propose methods of building best practices for patient involvement (PI) in HTA.

**Design:**

Throughout the process, documents were collected and participant observations were made. Individual interviews were conducted with patients, health‐care professionals and the INESSS scientific team, from January to March 2018.

**Results:**

Three committees were established: an expert patient committee to reflect on patient experience literature; an expert health professional committee to reflect on medical literature; and a co‐construction committee through which both patients and health‐care professionals contributed to develop the recommendations. The expert patients validated and contextualized a literature review produced by the scientific team. This allowed the scientists to consider aspects related to the patient experience and to integrate the feedback from patients into HTA recommendations. The most important factor contributing to a positive PI experience was the structured methodology for selecting patient participants, and a key factor that inhibited the process was a lack of training in PI on the part of the scientific team.

**Conclusions:**

This experience demonstrates that it is possible to co‐construct recommendations, even for technically complex HTA subjects, through a more democratic process than usual which led to more patient‐focused guidance.

## 1 INTRODUCTION

Patient involvement (PI) is gaining prominence in the area of health technology assessment (HTA). Policymakers, health‐care managers and professionals, and researchers are increasingly interested in users' experiences regarding the use of medical devices. In the field of research and evaluation, PI refers to doing research ‘with patients’ as opposed to ‘about patients’.^1^ User's experiential knowledge has recently been included in HTA, under the assumption that consideration of such information may lead to more relevant, humanistic and comprehensive consideration of the impact of new technologies on quality of life.^2^, ^3^ Scholars agree that PI in HTA should be undertaken on a case‐by‐case basis.^4^ Different frameworks have incorporated PI, ranging from a passive role, such as receiving information during medical encounters, to being actively involved in the co‐design and co‐construction of health‐care–related issues and guidance.^5^, ^6^ The latter considers the patient's experiential knowledge as an invaluable complement to scientific and academic expertise; it relies on the idea that patients and health‐care professionals can and should collaborate in the co‐construction of medical guidelines and recommendations (Table 1).^6^, ^7^

**Table 1:** Levels of patient involvement^6^

Level	Definition	Exemplary methodology
Consultation	Approach to obtain the perception, opinion and expertise of patients in order to explore a subject	Questionnaires, surveys, interviews, discussion groups
Collaboration	Approach by which patients are required to provide their point of view for the carrying out of a project	Work groups, patient expert committees
Co‐construction	Simultaneously engaging patients and professionals, based on the complementarity of each other's expertise and experiential knowledge, in order to carry out a joint activity from a common understanding	Joint expert committees (including professionals and patients)

Despite a general trend towards increased PI in HTA agencies that are members of the International Network of Agencies for Health Technology Assessment (INAHTA), PI remains limited in scope and highly variable in practice.^8^ In fact, various attempts to define models to better include patients in HTA processes have been made by HTA agencies worldwide. For example, the National Institute for Health and Care Excellence (NICE) in the United Kingdom promotes fairness and inclusion of patients in decisions related to their health and well‐being in its explicit public commitment policy.^4^ For this reason, NICE prioritizes HTA that includes patients with (a) experience of having a given condition and receiving care for it, (b) perception of the impact of the technology and (c) expectations about the technology's risks and benefits.^9^ A recent study in HTA research, designed to follow the PI standards described by NICE, found that while PI was overall a positive experience, patients valued their involvement at early stages of health technology development more, because they perceived that their contribution was greater. In the face of this evidence, the authors speculated that as patients are experts in their own illnesses, their contribution to the early development stage was key to better understanding patient needs and producing devices that responded to these needs.^10^ Another study assessing NICE's processes in incorporating the views of patients in HTA decision making found that although the organization has attempted to be flexible in integrating patient views, the patient's role is still confined to representation, rather than decision making.^11^

The Canadian Agency for Drugs and Technologies in Health (CADTH) also involves patients, on working groups and committees at several levels, in the assessment process for medications and medical devices, through the use of questionnaires.^12^ For example, the Pan‐Canadian Oncology Drug Review, an evidence‐based cancer medication assessment that is part of the programmes and services led by CADTH, offers patients the chance to share their experience as participants in clinical trials^13^, ^14^ and to describe needs unmet by current therapies.^12^

Likewise, elsewhere in Canada, Health Quality Ontario created the Ontario Health Technology Assessment Committee to provide guidance and advice on how to include PI in their HTA activities. This led to recommendations fostering PI in different forms. Moreover, a framework depicting PI in HTA was developed to support these initiatives.^15^ Although this framework is broad and all‐encompassing, it does not provide guidance regarding the selection of patients nor their means of participation in the different steps of the HTA process.^15^, ^16^, ^17^

Since 2010, INESSS has had an important role in the delivery of health care and social services to Quebeckers.^18^ The mission of INESSS is to promote clinical excellence by optimizing the use of resources when considering the incorporation and utilization of devices, medications and interventions. Thus, health‐related innovations entering the market are initially assessed; based on the results, INESSS issues recommendations concerning their utilization and implementation, as well as potential reimbursement of health‐care costs to users. Until the beginning of the present project, INESSS assessed products and services by consulting health‐care professionals, administrative managers and decision makers (usually recruiting the latter following consultation^19^); no patients were included in the co‐construction process of guidance, although a few evaluations were completed that included patient consultation.^20^ Moving forward, INESSS has adopted a 2016‐20 strategic plan looking to enhance the participation of knowledgeable users.^21^

Following the adoption of the 2016‐20 strategy^21^ and the 2016‐19 triennial activity plan,^22^ INESSS decided to include patients, along with health‐care professionals, in the co‐construction of recommendations concerning devices, medications and interventions.^6^ In the present manuscript, we describe the process of co‐construction of recommendations concerning the replacement of implantable cardiac defibrillators (ICDs) from the perspectives of both patients and health‐care professionals. ICDs are indicated for arrhythmia which is a cardiac disorder characterized by irregular or abnormally rapid or slow heartbeats. ICDs are devices that are placed under the skin to monitor heart rhythm and to intervene with electrical stimulation or shocks, as necessary, if arrhythmia occurs. Ventricular tachycardia and ventricular fibrillation are ‘malignant’ arrhythmias that can lead to sudden cardiac death,^23^ particularly in the presence of low left ventricular ejection fraction (defined as <40%)^24^, ^25^; for this reason, left ventricular dysfunction is a major criterion for the implantation, and continuing use, of ICDs. At present, ICDs require a replacement of their battery every 5‐7 years. During this period, the patient's clinical condition and treatment preferences may change. Moreover, ICDs can have significant impact on the daily lives of the people who wear them. On the one hand, they can prevent sudden cardiac death and provide a sense of security to the person, but on the other hand, shocks can be distressing and unexpected. It is therefore important, as with other implanted devices, that the patient or his/her representative be involved in the decision‐making process regarding initial implantation of an ICD and its replacement.

Following the publication of the ICD replacement guidance by INESSS,^26^ the aims of the present manuscript were to contribute to the discussion of PI best practices in HTA, by presenting the co‐construction methodology used and the learning experience of those involved in the process. A final objective is to propose a methodology for PI.

## 2 METHODOLOGY

### 2.1 Aims of data collection

The aims of the data collection carried out for the present project were firstly to continuously improve the co‐construction methodology process while it took place and secondly to assess the added value of PI to HTA,^27^, ^28^, ^29^ as appreciated by the various participants involved. Using a formative evaluation approach, we qualitatively assessed the feasibility and acceptability of our process, during which patients and health‐care professionals actively collaborated.^30^, ^31^ We combined data collection, logical analysis and implementation of changes^31^ in this project.

### 2.2 Informants

Four types of participants were queried: the patients (n = 8) and the health‐care professionals (n = 11) who were part of the co‐construction process, INESSS scientists (n = 4) and INESSS specialists in PI and ethics (n = 3). Prior to participating on the various committees, all persons external to INESSS received information about the purpose and rationale of the evaluation of ICD replacement.

### 2.3 Data collection

We followed the procedures of data collection for a formative evaluation by Brouselle and colleagues.^31^ Data were collected throughout the evaluative process and consisted of the following:

#### 2.3.1 Document collection

All documents produced during the project (eg minutes of meetings documents prepared for meetings, questionnaires and feedback forms from both patients and health‐care professionals) were collected to assist with assessing the co‐construction process.

#### 2.3.2 Participant observation

The first author (MPP) attended all meetings with the INESSS scientists and led all the committee meetings, as well as the co‐construction meeting, during which all experts (patients and health‐care professionals) were gathered. After each committee meeting, the first author (MPP) completed a logbook specifically designed for recording observations on the patients' and health‐care professionals' involvement. In total, 8 hours of meetings were held.

#### 2.3.3 Semi‐structured individual face‐to‐face interviews

In total, 23 interviews were conducted: 12 with patients (soon after their participation in both the patient (n = 6) and the co‐construction committee meetings (n = 6)), two with health‐care professionals and nine with INESSS staff members. All interviews were conducted by professionals trained in evaluative research (MPP and IG), from January to March 2018. Interviews varied in length from 30 to 90 minutes and were all digitally recorded; the topics discussed are presented in Appendix S1.

### 2.4 Data analysis

Each interview was transcribed by one member of the research team (MPP). Transcripts were imported into QDA miner software^32^ for coding purposes. Data analysis was carried out concurrently with data collection to allow the integration of information from each step of the process. We used the framework approach to analyse data,^33^ a strategy frequently used in the context of policymaking. This strategy employs five analytic stages, namely: (1) familiarization with the data through reading; (2) identification of a thematic framework that reflects the ideas discussed; (3) indexing data, that is identifying patterns across the transcripts; (4) charting data, that is comparing data across identified patterns; and (5) mapping and interpretation of data, that is making sense of the data as a whole. A logbook was kept to ensure the reproducibility of the analysis.

After each test of the PI process, internal reports were shared with INESSS staff members to discuss methods of improvement; a final report sent to those involved in the evaluation of the process was unanimously confirmed by all. In addition, in May 2018, we presented our findings at both a CADTH meeting^34^ and an INESSS Forum,^35^ during which we profited from feedback from the HTA community on which dimensions of our work were the most interesting for others in the field.

## 3 RESULTS

In this section, we present the four steps that were carried out to involve patients and to assess the PI process, as well as the perceived added value of our process according to others in the HTA community.

### 3.1 The choice of level of PI

At the beginning of its mandate, the INESSS scientific team, in collaboration with INESSS specialists in PI, aimed to develop a PI strategy to support the organization's initiative to develop the recommendations through co‐construction with patients. Firstly, a literature review on patient experiences, involvement in decision making and quality of life with respect to ICDs and particularly their replacement was conducted by the INESSS scientists. Information research strategies, adapted from a prior systematic review of high quality,^36^ were used and applied to the PubMed, EBM Reviews and EMBASE databases (Table 2).

**Table 2:** Strategies used to search the patient experience literature

Quality of life	The most recently published systematic reviews, of good quality according to the evaluation tool AMSTAR^37^ (January 2010‐March 2017)
Decision making	Systematic review by Lewis et al,^38^ covering the period of publication from January 2000 to November 2013, and primary studies or systematic reviews published between January 2013 and March 2017
Best practices in shared decision making	Systematic review by Lewis et al,^38^ covering the period of publication from January 2000 to November 2013, primary studies or systematic reviews published between January 2013 and March 2017 and a key scientific statement among the most recent published since 2010

**Figure 1:**
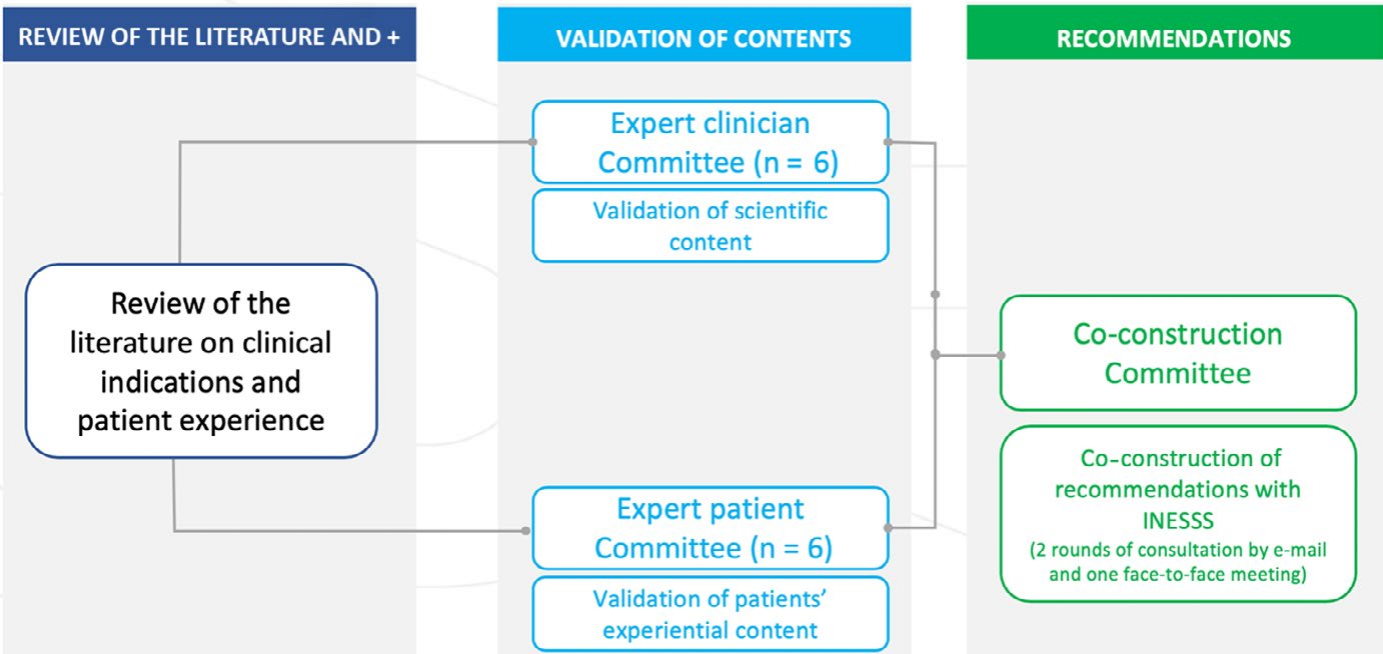
Process of involvement for the development of ICD replacement recommendations

The results of this literature search identified relevant 15 scientific articles. None of these were conducted in Québec. Following this step, the INESSS scientists and the PI team discussed which level of PI would be sought. The quantity, quality and content of the articles lead to the decision that the patients could be asked to participate in an analysis of the literature, just as expert professionals do. We thus created three committees: an expert patient committee to reflect on the literature concerning patient perspectives, an expert health‐care professional committee to reflect on the medical literature and a co‐construction committee for patients and health‐care professionals to contribute to the development of the recommendations together (Figure 1).

The mandate of the expert patient committee was to (a) share their experience of care and quality of life with an ICD; (b) discuss the available literature regarding patient decision making at the time of ICD implantation, replacement and deactivation (ie ceasing treatment by the device), (c) identify aspects of decision making and quality of life that would be important to be considered at the time of replacement, (d) discuss issues regarding optimizing the pathway of care for ICD patients and (e) contribute to the deliberation process leading to the recommendations (Table 3).

**Table 3:** Details of meetings

Meeting dates	Objectives and description	Participating committee
7 February 2017	First meeting of the committee, on the validation of the literature in relation to shared decision making	Expert patient committee
14 June 2017	Second meeting of the committee, on the validation of the literature in relation to quality of life	Expert patient committee
12 September 2017	Conference call to present the process for deliberation of recommendations	Expert patient committee
29 September and 7 November 2017 (dates of send‐out of recommendations)	2 rounds of a modified Delphi process regarding the recommendations by electronic mail	Co‐construction committee (expert patient committee AND expert health professional committee)

### 3.2 The process to recruit the expert patients

Recruitment of patients for the expert patient committee employed a methodology developed by Pomey and colleagues^6^ that recommends 4 steps, including (1) select those who respond to a general set of competency criteria (Table 4); (2) recruit using a team made up of a patient recruiter and a qualified person in charge of ‘partnership’; (3) train the patients in PI, partnership and guidance co‐construction; and (4) provide peer support to the recruited participants throughout the process by other patients who have already been trained or have participated in such a project.

**Table 4:** Patient selection criteria

From the University of Montréal^39^	For this specific project
Expresses him/herself clearly and simply Expresses general health network concerns through a constructive attitude about his/her treatment Has significant life‐experience with the condition under study Has significant experience in health care and services targeted by the project (see the criteria for this specific project) Is in a stable state of health at the time of recruitment (not in an acute or crisis situation) Has the ability to share his/her own experience with ICD use and has learned to live with it Can generalize his/her own experience to other contexts of care Demonstrates a desire to help people and contribute to an objective that goes beyond his/her individual experience Has interpersonal skills to facilitate collaboration (listening, empathy, etc) Has a critical mind, even within teams in which he/she has already been a patient Understands the vision and implications of the ‘partnership in health care’ of the Montréal model Is available and motivated to commit for the duration of the project	Living in various areas of Québec Treated by various health‐care institutions in Québec Having various diagnoses Wearing an ICD for various durations Having experienced a varying number of replacements (none to two) For at least some of the patients, having had previous experience of shock

We approached health‐care professionals at several health‐care institutions that were conducting ICD replacement to identify patients who might be interested in being part of the committee. Eight patients were referred to us through this mechanism. In addition, 3 more were identified by INESSS scientists through INESSS's involvement in a parallel field evaluation on ICD replacement. Thus, a total of 11 patients potentially interested in joining the committee were referred. Among these, 8 were contacted by a duo made up of the patient recruiter (SB) and a qualified person in charge of partnership (MPP). All 8 were selected according to specific criteria developed at the University of Montréal and those established for this particular project (ie ICD patients with different profiles) (Table 4). The final group was composed of 1 female and 7 males (reflecting the sex distribution of ICD users, the majority of whom are male), one person who had not had an ICD replacement, 6 people who had 2 replacements and one person who had experienced 3. Age varied from 45 to 83, with an average of 72 years. All expert patient committee members were paid for their time (except for one who requested to be a volunteer), like the experts on the health‐care professional committee). For their part, the health‐care professionals were selected to represent each of the ICD implanting centres and diverse domains of expertise; two professional societies of cardiologists in Québec were also involved in guiding this selection and both women and men took part.

### 3.3 The initial meetings and ways to mobilize the patients' expertise

Two meetings with the expert patient committee were organized by the INESSS scientists, prior to which a draft of the patient literature consulted and a list of questions related to this information were sent to each member. During the first hour of the initial meeting, the PI team talked about the reason why PI in HTA is relevant and presented the different methodologies available to involve patients in HTA (ie consultation, collaboration and co‐construction).^5^, ^6^ The goals of the patient committee meetings were to validate the knowledge base for the specific context of Québec regarding (1) decision making at the time of ICD replacement and (2) quality of life of patients living with an ICD. In parallel, the expert health‐care professional committee validated the medical scientific literature on ICD replacement.

### 3.4 The co‐construction of guidance

A modified Delphi method was subsequently carried out with the members of the joint committee through two consultation rounds by electronic mail.^40^ Comments and agreement regarding proposed wording for recommendations on ICD replacement were collected from the patients and health‐care professionals. This feedback was shared anonymously with all participants in the second round. The revised recommendations and the comments from the Delphi rounds were then presented during the in‐person co‐construction committee meeting to discuss: (1) the recommendations that engendered the most comments related to shared decision making and (2) the possibility of creating a tool to facilitate the decision‐making process between patients and physicians. The latter discussion resulted in a consensus between patients and health‐care professionals, with both groups favouring creation of a tool that would present the various treatment options according to the pathology involved, allowing patients to understand their illness and treatments, but without forcing patients to be included in decision making that could be particularly difficult, especially when the risk of mortality could be high.

### 3.5 The perception of the expert patients on their involvement

After each expert patient committee meeting, patients mentioned that these allowed for a discussion that helped them validate and contextualize the literature review, especially the sections concerning the patient's experience. They shined light on the themes they considered the most important for them and their loved ones. The patients highlighted the importance, at the time of the initial implantation, of recognizing the psychological impact of learning simultaneously that he/she was suffering from a dangerous, life‐threatening cardiac disease and that he/she was in urgent need of wearing an ICD. They also highlighted the impact on their family and loved ones, underlining the need for information to be given to these people as well, should they have questions. The patients all found it difficult to discuss the possibility of disabling the ICD. They all felt it was important for this topic to be addressed, but preferably after first reaching an understanding of their illness and the impact of having an ICD on their lives. Patients also added that information should be given by their treating physician or by a nurse whom they trust, and at the right time: when they are able to receive and integrate it.

The patients pointed out the need to better understand the organization of services, the role of each health‐care professional involved in their care and the way professionals communicate with each other. They mentioned the heterogeneity in the care pathway that leads to an ICD implantation. They also realized that the role of each health‐care professional can change from one patient to another: some patients are treated by a cardiologist and some by a general practitioner. It was also stated that more effort should be put towards promoting better communication between the different professionals involved, particularly before a replacement, to ensure all the technical information regarding the device is discussed.

The patients also mentioned the need for health‐care professionals to initiate a discussion about ICD generator replacement or deactivation with their patients and to maintain responsibility for the final decision. One reason for this was that patients felt they had received little information about the potential side‐effects of the ICD and the impact it would have on their daily activities, forcing them to go to the Internet to read on the matter. The second reason was that the replacement, for them, was not an ‘option’, since their ICD was presented to them as a long‐term treatment without any alternative: they felt the decision was to be made by their cardiologist or electrophysiologist.

The patients all learned for the first time during the meetings that the indication for an ICD could change over time. In the light of this, they felt it would be relevant to talk about the possible renewal of the ICD one year before the deadline for replenishing the battery support. This would help them to take part in the decision‐making process, especially if their treating physician was open to answering all of their questions and to take into consideration their desired level of involvement in their course of treatment. Although the patients expressed discomfort when presented with the possibility of deactivating the defibrillator function of their ICD, they underlined the importance of considering the patient's opinion and perspective, because of the psychologically reassuring effect of wearing the ICD. Indeed, during the expert health‐care professional committee meeting and the subsequent interviews, the clinicians agreed with the patients' perceptions that they were not involving their patients enough in the decision‐making process and did not have the necessary tools. All the concerns raised and noted above by the patients were incorporated into 7 of the 11 recommendations (Appendix S2).

### 3.6 The perception of the added valued of PI by the INESSS scientists and by the expert health‐care professionals

For the INESSS scientists, the contribution of the patients was evident at several levels. First of all, it allowed most of the team members to be in contact with patients with cardiac problems, which really brought home the point that there were people behind the statistics. The fact that INESSS reports could be read by patients became more obvious, and thus, the importance of paying attention to how information is presented:

I have been working in cardiology for more than 10 years and have never had the opportunity to meet and work directly with patients with heart problems. It made me a little nervous. I then realized that I had never imagined that reports written by INESSS could be read by patients. I will be more careful from now on. Now I'm paying a lot more attention to how I write my reports so the information is not too disturbing for patients.

This also allowed the team to be comfortable with several recommendations directly related to the patient experience:

[Probably if the patients had not co‐constructed the recommendations, we would not have had so many directly related to their experience with the renewal of an ICD. We would certainly have had strictly medical recommendations.]

For the health‐care professionals, they realized that they did not include patients fully in decision making and that the organization of services was not always optimal:

[We do not currently use tools to discuss different treatment options with patients. That's a good point.]

[I did not realize that patients were asking themselves so many questions about their daily life in relation to their ICD, such as going through security at the airport.]

[Indeed families are very minimally included throughout the process, as well as the attending physician.]

[The consent for the ICD change is actually made the same day as the operation; it is a good idea to consider it in advance.]

### 3.7 Factors that facilitated or inhibited PI

According to the INESSS scientific team, the most important factors that inhibited the PI process were their lack of previous work with patients and their inexperience using lived knowledge from patients in their prior assessments. For the INESSS scientist team, it was difficult, after the first meeting, to see the added value of the patient comments regarding the literature. Rather, they had been expecting data on the personal experiences of patients living with an ICD: ‘I did not learn anything new at the meeting. I thought we would mostly listen to their experience’. This remark was also made by the patients, as they would have all preferred to have had time at the beginning of the initial meeting to describe their experiences living with an ICD, issues that the scientists might not have previously appreciated:

[I would have liked to have devoted the initial meeting time to learning each other's stories. We learned these as we went along but it would have helped to better understand if other people had the same problem as me or not, and therefore if we had gone through similar experiences for the same reasons.]

The INESSS scientist team also pointed out that some of the decisions made to summarize the literature did not fully address all of the concerns of the patients. Indeed, patients highlighted the importance of including relatives in decision‐making discussions whereas this issue was not developed in sufficient depth in the initial text submitted to the committee, although it had been referred to by some of the scientific articles. Another challenge was related to how to integrate the perspectives of the expert patient committee into the report: after each of the three sections (quality of life, decision‐making experiences and best practices in decision making) should there be some kind of summary of the patients' input? A discussion between the scientific and PI teams led to the introduction of boxes summarizing the comments received, for the first time in an INESSS report.

The factors that most facilitated the patient recruitment process were the implementation of a structured approach to patient selection and the presence of a duo made up of a patient recruiter and a qualified person in charge of partnership to select, train and coach patients. This approach allowed the participants to benefit from the experience of another patient (the patient recruiter), in order to interact optimally throughout the process. This structure was very appreciated by the patients:

[We had a group where each person was relevant to the discussion],

[The discussions were very rich, I learned a lot about my illness and how ICDs work],

[I really appreciated that the recruitment was done by another patient].

As for the health‐care professionals, they appreciated the transparency of the process which reduced their uncertainty about having patients act as partners, rather than mainly as advocates: ‘[I was not sure what to expect, but I was pleasantly surprised to see patients being able to react to complex issues and give us as many solutions as possible to improve the organization of services]’.

## 4 DISCUSSION AND A PI FRAMEWORK PROPOSAL

This study has two main limitations. The first relates to the difficulty interviewing the persons on the expert health‐care professional committee. Only two of the 6 clinician experts who were present during the co‐construction committee meeting provided feedback on their experience, despite multiple efforts to contact them. This is probably best explained by a lack of availability on the part of the clinicians, but may also signal less interest in taking part in this kind of reflection. The second limitation concerns the fact that many of the authors of this article work for INESSS, which could lead to difficulty in being objective in the evaluative process.^31^, ^41^ On the other hand, this study made it possible to obtain detailed information about the various mechanisms that were used and the points of view of the actors in real time; the interviews seem to show that participants spoke freely.

The main goal of this article is to describe a co‐construction process to develop recommendations including both patient‐based evidence (PBE) and patient input.^42^ This level of PI in co‐construction is still rare in the literature: in fact, it is almost non‐existent.^2^, ^3^, ^4^, ^8^, ^10^, ^11^, ^12^ The aim was to test the ability of an HTA agency to advance PI by co‐constructing recommendations for ICD replacement with health‐care professionals and the INESSS scientific team. The methodology used shows that this is possible, even if the subject is complex as was the case for replacement of ICDs. This project shows that it is possible to engage patients in a ‘meaningful’ way, as proposed by Abelson et al (2018). Indeed, this approach is inscribed in the values of INESSS,^21^, ^22^ appreciating the experiential knowledge of patients, rather than a purely advocacy role, and it followed a structured process that defined what was expected by the level of chosen commitment. This has led INESSS to propose methods to build best practices for PI in HTA. In other words, INESSS took part in a sequential process that sought to identify a potential framework to be applied in future HTA processes of co‐construction, including both expert patients and health‐care professionals. In this context, the different steps that facilitated or inhibited the patients' involvement in the production of recommendations will be discussed below, as well as other issues that were raised during this project.

First of all, this project highlighted the importance of exploring patient‐related literature in order to evaluate the necessity of collecting primary data or asking patients using a health technology for their points of view. Indeed, when the scientific literature is not rich enough with respect to quality of life, shared decision making or patient empowerment, it is possible to consider adding a process of primary data collection among patients through consultation. This can be carried out through questionnaires, interviews or group discussions. When there is ample scientific literature, patients can be mobilized to reflect on it. However, as the patients and the INESSS scientific team realized, an important starting point is going around the table to discuss each patient's experience with the health condition(s) under study.

This project also highlighted the importance of clearly identifying the type of patients being sought to fulfil the mandate: in this case, patients with different pathologies requiring an ICD. The rigorous process of patient selection made it possible to set up a committee of patients with a variety of backgrounds and complementary expertise. To be able to efficiently identify patients with the required profile in the future at INESSS, it is necessary to (1) count on the support of health‐care providers and INESSS personnel to identify potential participants, (2) take advantage of different web platforms for the public such as the INESSS website, INESSS's Facebook page and Twitter, and (3) create a directory of patients who can be quickly mobilized to participate in INESSS's projects and can cover different demographics (age, gender, etc) and disease groups.

In addition, this project demonstrated that health‐care professional experiences in the field are also highly valued. INESSS made great effort to bring patients together with the scientific team to share their experience and expertise. Health‐care professionals too should be asked to share their clinical experience within committees. To optimize the participation process for patients and health‐care providers, some further structuring of INESSS's methods is warranted; to this end, an ethical framework for the integration of knowledge in health and social services (CRÉDIS),^43^ now in development, will be helpful.

Indeed, INESSS's scientific team was not trained in PI in HTA before the start of this project, which may have led to misunderstandings about how to carry out a co‐construction process. Consequently, a new partnership with the Centre of Excellence on Partnership with Patients and the Public (CEPPP),^44^ an academic centre specialized in PI, will make it possible to train all INESSS professionals, as well as patients and health‐care professionals involved in HTA, thus enhancing the contribution of patient participation and integration of experiential knowledge into INESSS recommendations^44^ in the future.

**Figure 2:**
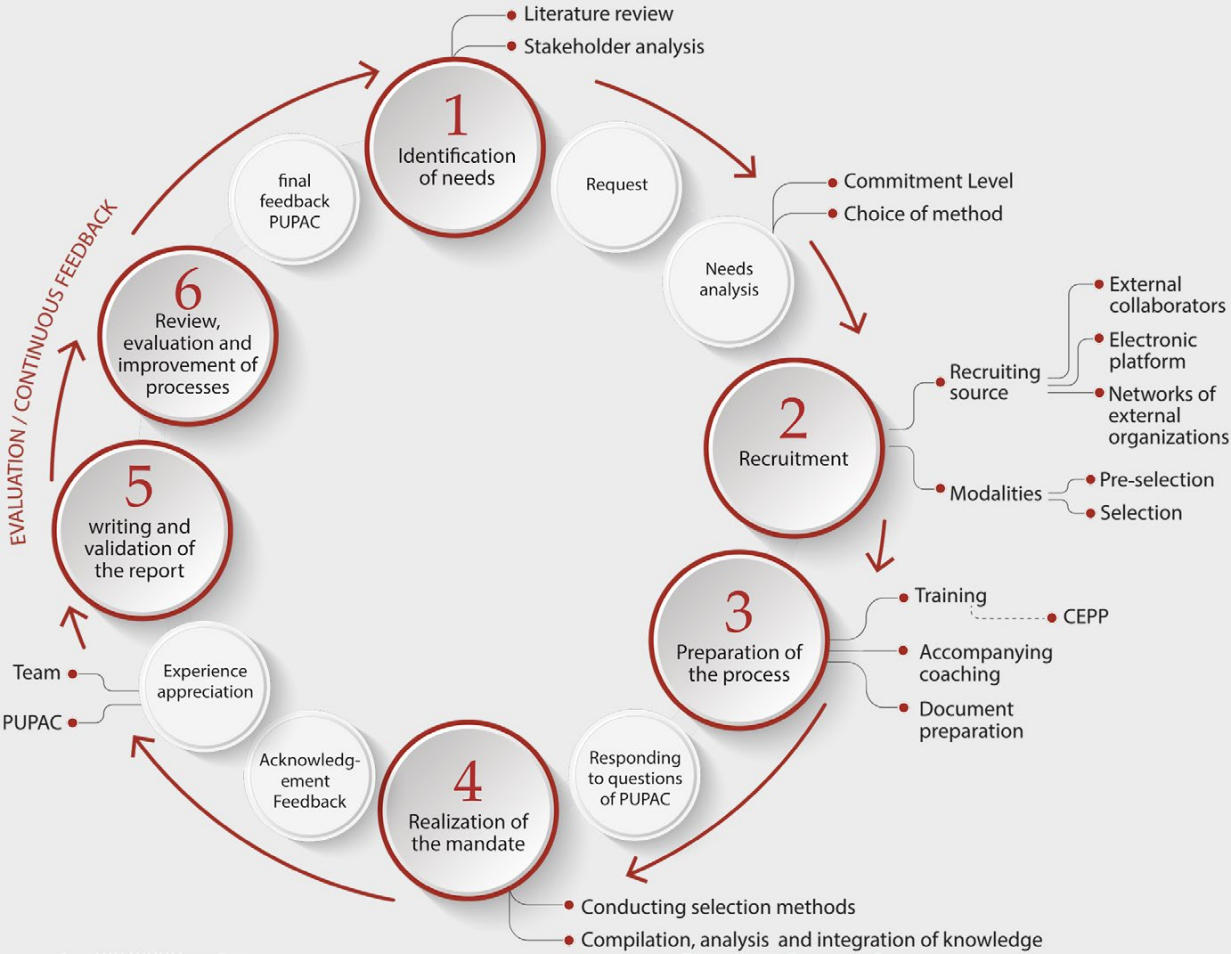
Process of patient involvement for the development of scientific products at INESSS

We also made sure that the participation of the members of the patient committee was acknowledged, firstly by thanking them individually not only by telephone and by e‐mail, but also by including their names in the reports that were published as a result of their work, with their permission. We ensured that lessons learned from the experience were translated into action to improve the process of developing HTA advice and practice guidelines. As a result of this project, a dedicated team, comprised of three partnership experts and two patients, was put in place to support the team methodology of patient participation in HTA, and a PI framework was adopted (see Figure 2).

Importantly, this project raised some ethical issues. Ethical questions concerned the following: whether it is possible to have patients and health‐care professionals who are in a clinical relationship on the same committee; how to respect the confidentiality of patient medical data held by INESSS; how to identify and adequately manage patients' conflicts of interest, by acknowledging that patients involved as experts in the development of recommendations related to their condition are not necessarily in conflict of interest for this reason; how to best include vulnerable patients, whatever those vulnerabilities might be; and how to guarantee a transparent methodology throughout the process. These issues and others have been the subject of reflection and resulted in a drafting of guidelines on various ethical issues related to patient participation at INESSS.^43^

New processes have also been proposed. For example, it has been suggested that organizing an earlier meeting between patients and the INESSS scientific team would have been useful, as the scientific team would then have benefitted from being made more aware of the importance of qualitative studies related to life‐experience with illness and decision making, and patients would have gained a better understanding of the scientific issues related to their health condition. Another proposal has been to identify one or two patients to support INESSS scientific teams in the definition of evaluation questions and in the interpretation of the patient experience literature, even before participating in a committee, as is already done for the scientific literature with key clinical experts.

Finally, despite a long tradition of using patient advocacy groups in Québec as compared to other jurisdictions, patients in this study were not recruited through patient associations because there were none for this specific health condition. For other projects, however, it would be interesting to see how it might be possible to unite both patients from an advocacy group and those living with a particular illness.

## 5 CONCLUSION

This project, conducted at INESSS, contributes to future development in optimizing the incorporation of PI in HTA through a co‐construction process, perhaps most importantly when the topics are complex. The evaluation of PI in the assessment of ICD replacement thus highlights the strengths and areas for improvement, as well as the challenges that have been encountered. INESSS strives to continue improving its methods to engage patients in partnership with health‐care professionals in a more systematic way throughout the entire assessment process, even if time constraints are a limiting factor. This study shows that it is possible to carry out co‐development of recommendations that combines patient and health‐care professional experiences. This allows a more democratic process than usual and contributes to a real commitment to PI in HTA, which results in recommendations with more patient focus and ultimately more potential impact on health services delivery and population health. Through this project, INESSS was able to propose a framework for meaningful PI in its various activities, a framework that could also help other HTA agencies to better structure their own approach and promote optimal IP in their evaluation work.

## CONFLICT OF INTEREST

The authors declare that there is no conflict of interests.

## Data Availability

The data that support the findings of this study are available in [INESSS] at [https://www.inesss.qc.ca/fileadmin/doc/INESSS/Forum_metho_2018/Session_3_Ganache_Lambert_Bedard.pdf], reference number [32]. These data were derived from the following resources available in the public domain: [https://www.inesss.qc.ca/en/publications/documents-methodologiques/forum-methodologique-2018.html].

## References

[1] NIHR . National standards for public involvement in research. March 2018 https://www.invo.org.uk/wp-content/content/content/uploads/2019/02/71110_A4_Public_Involvement_Standards_v4_WEB.pdf. Accessed February 27, 2019.

[2] Mamzer M‐F , Dubois S , Saout C , et al. How to strengthen the presence of patients in health technology assessments conducted by the health authorities. Therapies. 2018;73(1):95‐105. 10.1016/j.therap.2017.11.004 29478707

[3] Facey K , Hansen HP , Single A . Patient Involvement in Health Technology Assessment. ADIS; 2017 https://www.springer.com/gp/book/9789811040672

[4] Weeks L , Polisena J , Scott AM , Holtorf AP , Staniszewska S , Facey K . Evaluation of patient and public involvement initiatives in health technology assessment: a survey of international agencies. Int J Technol Assess Health Care. 2017;33(6):715‐723. 29122048 10.1017/S0266462317000976

[5] Carman KL , Dardess P , Maurer M , et al. Patient and family engagement: a framework for understanding the elements and developing interventions and policies. Health Aff (Millwood). 2013;32(2):223‐231. 23381514 10.1377/hlthaff.2012.1133

[6] Pomey M‐P , Flora L , Karazivan P , et al. Le « Montreal model » : enjeux du partenariat relationnel entre patients et professionnels de la santé. Santé publique. 2015;S1(HS):41‐50. 26414256

[7] Council of Europe . Civil Participation in the Decision‐Making Process, The Code of Good Practice; Kyiv, October 2009 https://rm.coe.int/CoERMPublicCommonSearchServices/DisplayDCTMContent?documentId=09000016802eede1. Accessed August 5, 2019.

[8] Hailey D , Werkö S , Bakri R , et al. Involvement of consumers in health technology assessment activities by INAHTA agencies. Int J Technol Assess Health Care. 2013;29(1):79‐83. 23217279 10.1017/S026646231200075X

[9] NICE . Guide to the methods of technology appraisal 2013. April 4, 2013 https://www.nice.org.uk/process/pmg9/resources/guide-to-the-methods-of-technology-appraisal-2013-pdf-2007975843781. Accessed February 27, 2019.

[10] Simpson S , Cook A , Miles K . Patient and public involvement in early awareness and alert activities: an example from the United Kingdom. Int J Technol Assess Health Care. 2018;34(1):10‐17. 29508684 10.1017/S0266462317004421

[11] Hashem F , Calnan MW , Brown PR . Decision making in NICE single technological appraisals: how does NICE incorporate patient perspectives? Health Expec. 2017;21(1):128‐137. 10.1111/hex.12594 PMC5750768 28686809

[12] CADTH . Patient involvement in scientific advice. https://www.cadth.ca/scientific-advice/patient-involvement. Accessed November 17, 2018.

[13] Rozmovits L , Mai H , Chambers A , Chan K . What does meaningful look like? A qualitative study of patient engagement at the Pan‐Canadian Oncology Drug Review: perspectives of reviewers and payers. J Health Serv Res Policy. 2018;23(2):72‐79. 29624087 10.1177/1355819617750686

[14] Berglas S , Jutai L , MacKean G , Weeks L . Patients' perspectives can be integrated in health technology assessments: an exploratory analysis of CADTH Common Drug Review. Res Involv Engagem. 2016;2:21. 29062521 10.1186/s40900-016-0036-9 PMC5611639

[15] Abelson J , Wagner F , DeJean D , et al. Public and patient involvement in health technology assessment: a framework for action. Int J Technol Assess Health Care. 2016;32(4):256‐264. 27670693 10.1017/S0266462316000362

[16] Gagnon M‐P , Desmartis M , Gagnon J , et al. Framework for user involvement in health technology assessment at the local level: views of health managers, user representatives, and clinicians. Int J Technol Assess Health Care. 2015;31(1–2):68‐77. 25952585 10.1017/S0266462315000070

[17] Abelson J . Patient engagement in health technology assessment: what constitutes ‘meaningful’ and how we might get there. J Health Serv Res Policy. 2018;23(2):69‐71. 29411660 10.1177/1355819618756936

[18] Loi sur l'Institut national d'excellence en santé et en services sociaux. In: Québec Gd , ed. I‐13.03. Québec: Éditeur officiel du Québec; 2010.

[19] INESSS . Institut national d'excellence en santé et en services sociaux. 2018 https://www.inesss.qc.ca/. Accessed November 18, 2018.

[20] Beaulieu M‐D , Pomey M‐P , Côté B , et al. Soutenir l'amélioration continue de la qualité des soins donnés aux personnes souffrant de maladies chroniques au Québec. Institut national d'excellence en santé et services sociaux (INESSS). 2012;8(12):1‐68.

[21] Institut national d'excellence en santé et en services sociaux (INESSS) . Plan stratégique de l'INESSS 2016‐2020. Montreal; 2017 https://www.inesss.qc.ca/fileadmin/doc/INESSS/Rapports/MaladiesChroniques/ETMIS2012_Vol8_No12.pdf. Accessed February 28, 2019.

[22] Institut national d'excellence en santé et en services sociaux (INESSS) . Plan triennal d'activités 2016‐2019. Québec; March 17, 2016 https://www.inesss.qc.ca/fileadmin/doc/INESSS/DocuAdmin/PTA_2016_2016-07-18.pdf. Accessed February 28, 2019.

[23] Bennett M , Parkash R , Nery P , et al. Canadian Cardiovascular Society/Canadian Heart Rhythm Society 2016 implantable cardioverter‐defibrillator guidelines. Can J Cardiol. 2017;33(2):174‐188. 28034580 10.1016/j.cjca.2016.09.009

[24] Adabag AS , Luepker RV , Roger VL , Gersh BJ . Sudden cardiac death: epidemiology and risk factors. Nat Rev Cardiol. 2010;7(4):216‐225. 20142817 10.1038/nrcardio.2010.3 PMC5014372

[25] Armstrong PW . Left ventricular dysfunction: causes, natural history, and hopes for reversal. Heart. 2000;84(Suppl 1):i15‐17:discussion i50. 10956313 10.1136/heart.84.suppl_1.i15 PMC1766527

[26] Azzi L , Boothroyd L , Collette C , et al. Les éléments facilitant la prise de décision dans le contexte du remplacement du générateur d'un défibrillateur cardiaque implantable. Montréal: Institut National d'excellence en Santé et Services Sociaux; 2018.

[27] Patton MQ . Developmental Evaluation Applying Complexity Concepts to Enhance Innovation and Use. New York, NY: Guilford Press; 2010.

[28] Abelson J , Humphrey A , Syrowatka A , Bidonde J , Judd M . Evaluating patient, family and public engagement in health services improvement and system redesign. Healthc Q. 2018;21(SP):61‐67. 30566406 10.12927/hcq.2018.25636

[29] Hailey D , Werkö S , Rosén M , et al. Influence of health technology assessment and its measurement. Int J Technol Assess Health Care. 2016;32(6):376‐384. 28124969 10.1017/S0266462316000611

[30] Brousselle A , Breton M , Benhadj L , et al. Explaining time elapsed prior to cancer diagnosis: patients' perspectives. BMC Health Serv Res. 2017;17(1):448‐448. 28659143 10.1186/s12913-017-2390-1 PMC5490154

[31] Brousselle A , Champagne F , Contandriopoulos A , Hartz Z . L'évaluation: concepts et méthodes. Montréal: Presses de l'Université de Montréal; 2011.

[32] Research P . Qualitative data analysis software, mixed methods research tool. https://provalisresearch.com/products/qualitative-data-analysis-software/. Accessed December 12, 2018.

[33] Ritchie J , Spencer L . Qualitative data analysis for applied policy research In: Huberman AM, Miles MB, eds. The Qualitative Researcher's Companion. Thousand Oaks, CA: Sage; 2002:305‐329.

[34] Lambert L , Boothroyd L , Brouillard P , et al. An Evaluation That Aims to Optimize the Shared Decision‐Making Process about Defibrillator Replacement: A Multi‐Method Approach. Montreal: CADTH; 2018.

[35] Bédard S , Ganache I , Lambert L , Pomey MP . Regards croisés: retour d'expérience sur la réalisation de l'avis sur le remplacement du générateur d'un défibrillateur cardiaque implantable In: INESSS, ed. Forum méthodologique de l'INESSS: INESSS. Montreal, QC:INESSS; 2018.

[36] Lewis KB , Stacey D , Carroll SL , Boland L , Sikora L , Birnie D . Estimating the risks and benefits of implantable cardioverter defibrillator generator replacement: a systematic review. Pacing Clin Electrophysiol. 2016;39(7):709‐722. 26969818 10.1111/pace.12850

[37] Shea BJ , Grimshaw JM , Wells GA , et al. Development of AMSTAR: a measurement tool to assess the methodological quality of systematic reviews. BMC Med Res Methodol. 2007;7:10. 17302989 10.1186/1471-2288-7-10 PMC1810543

[38] Lewis KB , Stacey D , Matlock DD . Making decisions about implantable cardioverter‐defibrillators from implantation to end of life: an integrative review of patients' perspectives. Patient. 2014;7(3):243‐260. 24668214 10.1007/s40271-014-0055-2

[39] Pomey M‐P , Lebel P , Clavel N , et al. Development of patient‐inclusive teams: Toward a structured methodology. Healthc Q. 2018;21(SP):38‐44. 30566402 10.12927/hcq.2018.25640

[40] Custer RL , Scarcella JA , Stewart RR . The modified Delphi technique—a rotational modification. J Career Tech Educ. 1999;15(2):50‐58.

[41] Patton MQ , Conrad RE . Qualitative Research & Evaluation Methods. Sage; 2002.

[42] Staniszewska S , Werkö S . Patient‐based evidence in HTA In: Facey KM, Ploug HH, Single A, eds. Patient involvement in health technology assessment. 1st ed Singapore: Springer Nature; 2017:43‐51.

[43] Goetghebeur M , Demers‐Payette O , Pomey MP , Ganache I , Roy D . Development of an institutional ethical framework for the evaluation of interventions in health care and social services for public coverage: a focus on stakeholder participation. CADTH Technol Overv. 2018.

[44] CEPPP . CEPPP: Together I Am Better. 2018 https://ceppp.ca/en/. Accessed November 18, 2018.

