# Supplementary material for: Co‐construction of health technology assessment recommendations with patients: An example with cardiac defibrillator replacement
Source: Health Expect. 2019 Nov 5;23(1):182–92. doi: 10.1111/hex.12989 (PMC6978850; doi:10.1111/hex.12989)
Supplement: Supplementary file 1 [file HEX-23-182-s001.docx]

Appendix S1

Topics covered during the individual semi-structured interviews.

(a) Patient interview:

- - Interest in participating in the work of INESSS;
  - The way their experiential knowledge was taken into account;
  - Preparation and organization of their participation (training, logistics, documents, etc.);
  - The co-construction meeting for the deliberation of the recommendations;
  - The impact of their participation on themselves and how to live with their illness;
  - How to improve the process.

(b) Health care professional interview:

- - The added value of the co-construction committee for deliberation of the recommendations;
  - The added value of the integration of a literature review that relates to the patient experience and feedback from a patient committee into the report.
  - How to improve the process.

(c) INESSS research team interview:

- - The patient selection process;
  - The PI methods;
  - The integration of patient knowledge into the guidance;
  - Patient input to the recommendations;
  - Lessons learned.
