# Supplementary material for: Co‐construction of health technology assessment recommendations with patients: An example with cardiac defibrillator replacement
Source: Health Expect. 2019 Nov 5;23(1):182–92. doi: 10.1111/hex.12989 (PMC6978850; doi:10.1111/hex.12989)
Supplement: Supplementary file 4 [file HEX-23-182-s004.docx]

Appendix 2

| 1 | Patients should be informed, upon initial ICD/CRT-D implantation, that when the time comes to replace the generator, a re-evaluation will be carried out to ensure that the most appropriate treatment is offered, and that different options will be available at that time. | Recommendation related to the patient |
| --- | --- | --- |
| 2 | The name of the treating physician (electrophysiologist, cardiologist, internist or family physician) should be clearly indicated at the time of initial ICD/CRT-D implantation and updated at follow-up visits, if necessary. | Recommendation related to the patient and communication between professionals |
| 3 | The location and mechanisms of follow-up should be indicated and clearly explained to the patient upon initial ICD/CRT-D implantation and should be modifiable according to changes in needs. | Recommendation related to the patient |
| 4 | Patients with an implanted ICD/CRT-D should have a follow-up at least every 6 months in person or remotely at or by the implantation centre and/or a clinic qualified to do so, and there should be an in-person meeting with the patient at least once a year. A follow-up at least every 3 months is recommended at the first indication of low battery. | Recommendation related to the patient and a medical recommendation regarding frequency of follow-up |
| 5 | When the remaining lifespan of the generator battery is estimated to be less than 1year, the team providing the ICD/CRT-D follow-up should inform the treating physician in writing so that a re-evaluation of the patient’s overall condition can be planned. This communication should include the following: the type of device; an easy-to-understand follow-up report on the ICD/CRT-D that includes the history of therapy provided by the device since the last intervention; the patient’s needs for cardiac stimulation. | Medical recommendation |
| 6 | Upon receiving the low battery warning, the treating physician should meet with the patient to record key information on a standardized form which is then sent to the implantation centre. This form should be available in the medical file when the patient comes to his/her ICD/CRT-D replacement/modification appointment and should include the following: the patient’s care objectives; the patient’s overall clinical condition; the patient’s NYHA functional class; a recent electrocardiogram (ECG) (width of the QRS complex, presence of left bundle branch block (LBBB), ventricular stimulation); the patient’s most recent left ventricular ejection fraction (LVEF), preferably determined in the past year. | Recommendation related to the patient and a medical recommendation (regarding clinical information to be recorded) |
| 7 | The clinical criteria in the Canadian practice guidelines for an initial ICD implantation should be taken into consideration when making a replacement decision. Therefore, maintenance of the defibrillator function is recommended if it is felt that the patient’s life expectancy can be significantly improved and if one or more of the following factors are present: LVEF ≤ 30%; history of malignant arrhythmia or cardiac arrest due to ventricular tachycardia or ventricular fibrillation; cardiomyopathy or a genetic anomaly associated with a risk of cardiac arrest or of malignant arrhythmia.  The defibrillator function can be maintained if the patient’s LVEF is between 31 and 35%, especially in the presence of symptoms of NYHA Class II or III heart failure.  Consideration can be given to not maintaining the defibrillator function if the patient’s LVEF is greater than 35% and he/she has not experienced a malignant arrhythmia (or received appropriate therapy from the ICD) since initial implantation. However, given that the risk of such arrhythmia is not zero, there should be a thorough discussion with the patient to ensure his/her understanding and respect of his/her wishes.  It should be determined whether the patient is a candidate for the addition of CRT. | Medical recommendation |
| 8 | The implanting electrophysiologist should inform the treating physician in writing of any procedure performed. | Recommendation related to the patient and communication between professionals |
| 9 | Each residential and long-term care centre, private or semi-private equivalent, or palliative care facility should have a protocol in place for identifying residents with an implanted ICD/CRT-D on admission, determining care objectives with the person (or his/her representative in the case of incapacity to consent to treatment) and recording the name of the facility responsible for ICD/CRT-D follow-up in the patient’s file.*.* | Recommendation related to the patient |
| 10 | Each residential and long-term care centre, private or semi-private equivalent or palliative care facility should have a protocol in place to ensure timely access to services for deactivating defibrillator function. | Medical recommendation |
| 11 | To facilitate discussion of care objectives with patients, universities and professional federations and associations should offer training on shared decision-making to health care professionals, and a provincial committee or equivalent should develop decision support tools in collaboration with multidisciplinary experts. | Recommendation related to the patient |
